# Prevalence and Circulating Serotypes of Dengue in Bastar, Chhattisgarh: A Cross-Sectional Study

**DOI:** 10.1155/cjid/7569212

**Published:** 2025-03-14

**Authors:** Rani Soni, Dhananjay Tandon, Sahina Hassan, Debashish Samal, Divakar Sharma

**Affiliations:** ^1^Department of Applied Science, Shri Rawatpura Sarkar University, Raipur 492016, Chhattisgarh, India; ^2^Department of Microbiology, Late Baliram Kashyap Memorial Government Medical College, Jagdalpur 494001, Chhattisgarh, India; ^3^Department of Biotechnology, Graphic Era (Deemed to be) University, Dehradun 248002, Uttarakhand, India

**Keywords:** dengue, IgM, NS1, prevalence, serotyping

## Abstract

The dengue virus is a significant re-emerging arbovirus drawing global public health concern. Urbanization, population growth, human mobility, water access, and storage practices contribute to its transmission. This hospital-based cross-sectional study is designed to determine dengue infection and prevalence in the district Bastar, Chhattisgarh. Blood samples were collected from the patients, and based on fever duration, they were tested for nonstructural protein 1 (NS1) antigen and immunoglobulin M (IgM) antibody detection. NS1 positive cases were further tested by RT-PCR for serotyping. Among the 2223 collected samples, 2041 were screened for NS1 and 182 for IgM; among them, the positivity was 55 (2.70%) in NS1 and 23 (12.63%) in IgM, respectively. Overall positivity of the dengue cases was 78 (3.51%); however, sex-wise, male and female, dengue positive cases were 45 and 33, respectively. NS1 was positive in 55 cases (70.51%), and IgM in 23 (29.49%) patients. Among these 78 cases, 4 NS1 and 2 IgM cases have shown symptoms of warning signs, while the rest of the cases have shown nonwarning symptoms. Among the 55 NS1 positive cases, the age group (21–60 years) was most affected by 45 (81.81%) DENV cases and the prevalent serotype was DENV-2 in singly and DENV-1 and DENV-2 in combination. The study's serotyping data might signify the early detection and identification of circulating serotypes, which provides valuable insights to clinicians for managing dengue infections. Hence, continuous epidemiological surveillance of DENV in the area is essential to anticipate future heterologous infections and their impact on healthcare. Early detection and vigilant monitoring of patients are crucial for identifying the circulating serotypes of dengue virus, facilitating subsequent epidemiological studies and disease control strategies.

## 1. Introduction

Dengue virus (DENV) is an emerging mosquito-borne arboviral infection endemic to urban and suburban areas of tropical and subtropical countries worldwide [1]. The Centers for Disease Control and Prevention (CDC) records cases of dengue imported into the continental United States annually [2, 3]. According to the WHO, dengue is classified into mild, moderate, and severe categories. Symptoms of mild dengue include no symptoms, while symptoms of moderate dengue include abdominal pain, persistent vomiting, fluid buildup, mucosal bleeding, tiredness, liver enlargement, and a higher hematocrit with a lower platelet count. Severe dengue is associated with complications such as severe plasma leakage, severe bleeding, or organ failure. Among the four DENV serotypes, DENV-1 has been identified as having a higher risk of severe dengue based on WHO criteria.

A DENV infection can manifest a broad range of disease severity, from influenza-like symptoms to life-threatening conditions like severe dengue, either with or without warning signs [4]. The virus belongs to the family *Flaviviridae* and the genus *Flavivirus*. It has four serotypes (DENV-1, DENV-2, DENV-3, and DENV-4), which have undergone genetic and antigenic variations due to mutations and resistance to host immune responses. Infection with a specific DENV serotype provides lifelong immunity against that serotype but does not confer protection against others [5]. DENV is a single-stranded, positive-sense RNA virus with a genome approximately 11 kb in length [6]. Globally, an estimated 390 million dengue cases occur annually, leading to approximately 500,000 hospitalizations and 25,000 deaths [7].

These revised guidelines provide clearer clinical criteria to ensure a standardized global approach to disease classification. DENV infections may be asymptomatic or symptomatic [8]. Symptomatic cases are categorized as dengue fever (DF), dengue hemorrhagic fever (DHF), or dengue shock syndrome (DSS) [9]. Undifferentiated febrile illness (UF) and DF account for the majority of symptomatic cases, while approximately 10% of cases progress to DHF/DSS [10].

In August 2015, the first dengue outbreak was reported in Konta, a remote tribal area in the Bastar district of Chhattisgarh, India. This outbreak affected a population of 7,038, but no severe dengue cases or fatalities were recorded. The absence of severe cases was largely attributed to an efficient mosquito control campaign implemented by the State Infectious Disease Control Program. The State Infectious Disease Control Program reported no dengue-like cases in the same area for two years. To prevent future dengue outbreaks, surveillance programs and dengue serotyping using RT-PCR are necessary during the monsoon and post-monsoon seasons. A study conducted in a remote tribal village in central India detected DENV-3 in 46.10% of patients, but no correlation was found between serotype and disease severity [11].

The Indian Council of Medical Research (ICMR) and the Department of Health Research have established Viral Research and Diagnostic Laboratories (VRDLs) across India to facilitate the diagnosis and research of viral diseases [12]. A study on the community transmission of a dengue outbreak was conducted in Bhilai, Chhattisgarh, in 2018. The survey assessed public awareness using questionnaires and found that excessive humidity and high ambient temperatures created ideal conditions for mosquito breeding, leading to increased transmission. A lack of awareness among the local population further contributed to the high rate of dengue cases [13].

Dengue remains a major public health concern in tropical and subtropical regions, particularly during the monsoon and post-monsoon seasons. Early detection of nonstructural protein 1 (NS1) antigen and immunoglobulin M (IgM) antibodies in the acute phase can aid in timely diagnosis. Community-based cohort studies are essential for reducing mortality rates by predicting the spread of dengue infections [14]. Considering the severity of dengue, the present study aims to contribute significantly to public health efforts to control and eliminate the spread of the infection. DF is now widespread in several high-endemic districts of Chhattisgarh, with Bastar being among the most affected regions. This study investigates the prevalence, mortality rate, and circulating serotypes among patients with a history of fever in a tertiary care hospital in the Bastar district.

## 2. Materials and Methods

### 2.1. Defining of Study Area and Time Period

The research was conducted in the Bastar district, situated on a plateau approximately 2000 ft above sea level of the southern region of Chhattisgarh, which is prone or endemic for DENV. The estimated total study area encompassed a 50-square-kilometer radius. The population of Bastar is approximately 9.56 lakh, which has dry, moist tropical, and forested regions. The temperature tends to be lower due to the shedding of leaves and the forest cover. Rainfall predominantly occurs during the southwest monsoon season, with the majority falling between June and September; however, rainfall in other months varies. On average, Bastar experiences 44.36 rainy days annually, with an average annual rainfall of 116.67 mm. The climate is characterized by humidity levels of 60.35% and an average temperature of 27.69°C. Annual precipitation averages 66.67 mm. The investigation was done from June to December 2023. Before initiating this study, ethical approval was taken from the Institute Ethics Committee with file number F.No./4051/GMCJ/Estt/2023 and consent was obtained from participating patients. Data collection from patients was carried out using a questionnaire.

### 2.2. Blood Sample Collection

Blood samples were collected from various collection centers, tertiary care hospitals in Bastar district, including primary health centers and community health centers, and medical camps organized by our medical college team. Blood samples were collected in sterile vacutainer tubes and transported to the virology laboratory in cold chain, and then the samples were centrifuged at 3000 rpm for 10 min at 4°C in order to isolate serum, which would be stored at −80°C for further analysis. A total of 2223 individuals were included in this study. The research was conducted at the Virus Research and Diagnostic Laboratory, Department of Microbiology, Lt. Baliram Kashyap Memorial Government Medical College, Jagdalpur, funded by DHR/ICMR, New Delhi. The data were categorized according to sex, age, and symptoms (fever, vomiting, weakness, body ache, peripheral shock, and bleeding during vomiting).

### 2.3. Inclusion Criteria

Patients with a range of ages between 0 and 65 years exhibiting DF with standard criteria, including the presentation of febrile illness lasting 2–7 days with symptoms such as headache, myalgias, arthralgias, rash, hemorrhagic manifestations, and leukopenia, were included in this study. Specimens negative for malaria and Japanese encephalitis virus (JEV) were also included.

### 2.4. Exclusion Criteria

Patients without a febrile illness for a specified period of time, as well as patients without typical dengue symptoms, were excluded. We also excluded patients with urinary tract infections, pneumonia, abscesses, or other obvious causes of fever.

### 2.5. Screening of Dengue Positive Cases by Presence of NS1 Antigen

The Panbio Dengue Early ELISA Kit (Abbott Diagnostic Korea Inc.) was utilized to detect dengue nonstructural protein (NS1) when fever was present for less than 5 days. Briefly, 100 μL of diluted blood samples, controls, and calibrators were placed into their respective micro wells as per the manufacturer's instructions. The wells were then covered with aluminum foil and incubated for 1 hour at 37°C. After the incubation period, each well was washed six times with 1x wash buffer. Next, 100 μL of HRP-conjugated Anti-NS1 MAb was added to each well and incubated for 1 hour at 37°C. The wells were washed three times with 1x washing buffer. Following this, 100 μL of TMB was added to each well and incubated for 10 min at 37°C. The test was terminated by adding 100 μL of stop solution, and the color intensity was measured at a wavelength of 450 nm within 30 min using the Erba Lisa Scan automated ELISA reader (Transasia Bio-Medicals Ltd., India). If the Panbio units are > 11 (cutoff), then it is considered as positive detection of the dengue infection. Panbio units are calculated by the formula: Panbio units = index value × 10 as per the kit protocol. Index value is calculated by another formula; index value = sample absorbance/cutoff value; here cutoff value is the mean of calibrators' absorbance × calibration factors (as per kit protocol).

### 2.6. Diagnosis of IgM Positive Dengue Cases

The presence of IgM was diagnosed when fever persisted for more than 5 days using the MAC ELISA kit (NIV Pune, India, Lot No. 23-023). In brief, 50 μL of diluted blood samples and controls were added to appropriate micro wells, covered with aluminum foil, and then incubated at 37°C for 60 min. After the incubation period, the wells were washed five times with a 1 × wash buffer. Subsequently, 50 μL of antigen was added to each well and incubated at 37°C for 1 hour. Following another round of washing three times with 1 × washing buffer, 50 μL of monoclonal antibody was applied and incubated at 37°C for 1 hour. The wells were then washed 3 times with 1x washing buffer. Next, 50 μL of the HRP conjugate was added to each well and incubated at 37°C for 30 min. We washed the wells three times with 1x diluted wash buffer after the incubation. Finally, 100 μL of TMB was added to each well and incubated at 37°C for 10 min. To stop the reaction, 100 μL of stop solution was added. Within 10 min, an Erba Lisa Scan automated ELISA reader (Transasia Bio-Medicals Ltd., India) was used to read the absorbance at 450 nm. If the optical density (OD) of samples exceeds the OD of the negative control by a factor of 3.0, it is considered as the cutoff value for the positivity.

### 2.7. Serotyping of the DENV Positive Cases by Molecular Approach (RT-PCR)

RT-PCR (Quant Studio 5, Applied Biosystems, Singapore) was used to confirm the DENV serotypes. This method involves turning RNA into cDNA backwards and then amplifying the cDNA with PCR. The presence of an amplified product indicates the presence of DENV RNA in the sample. We extracted viral RNA from NS1-positive serum samples using the Qiagen-QIAmp viral RNA mini kit, following the manufacturer's protocol. In brief, a reaction mixture was prepared in a 1.5 mL Eppendorf tube. In the first tube, 560 μL of AVL buffer and 5.6 μL of carrier RNA were combined. After that, 140 μL of the serum sample was mixed with 565.6 μL of AVL buffer containing carrier RNA. The mixture was vortexed for 15 s, and then it was incubated at room temperature for 10 min. Afterward, the samples were briefly centrifuged at 8000 rpm for 10 s to remove any droplets formed inside the tube cap. Next, 560 μL of absolute ethanol was added to the lysate, vortexed for 15 s, and briefly centrifuged. Then, 630 μL of the resulting solution was applied to a spin column and centrifuged at 8000 rpm for 1 min at room temperature. The supernatant was discarded, and the process was repeated. Subsequently, 500 μL of AW1 buffer was added to the spin column and centrifuged at 8000 rpm for 1 min at room temperature, followed by discarding the supernatant. Next, 500 μL of AW2 buffer was added to the spin column and centrifuged at 14,000 rpm for 3 min at room temperature. After discarding the supernatant, the spin column was centrifuged at 14,000 rpm for 1 min to completely dry the membrane. The dried column was then transferred to a 1.5 mL recovery tube. Subsequently, 50 μL of elution buffer was added to the center of the column, and the lid was closed. The column was incubated for 1 min and then centrifuged at 14,000 rpm for 1 min. Finally, the spin column was discarded, and the recovery tube contained the extracted viral nucleic acids. The viral RNA was stored at −20°C for further use.

The RealStar Dengue RT-PCR Kit 1.0 (Altona Diagnostics) was used to determine the serotypes of the samples that were positive for viral RNA. Master Mix preparation and serotype RT-PCR were performed as per the manufacturer's protocol. In brief, for DENV1-4, 5 μL Master Mix A (DENV 1 and 4) and 15 μL Master Mix B (DENV 2 and 3) were poured in an Eppendorf tube. The Master A and Master B sets each have everything you need to do reverse transcription, PCR-mediated amplification, detection of target-specific RNA, and internal control in a single reaction setup. These sets include PCR buffer, reverse transcriptase, DNA polymerase, magnesium salt, primers, and probes. 20 μL of the master mix was poured into each required well. Thereafter, 10 μL of extracted RNA was added into the respective micro wells and mixed. One positive and one negative control were also added in two separate wells. The plate was closed with optical adhesive film. We ran the reactions in Quant Studio 5 RT-PCR. The RT-PCR program was as follows. The program involved reverse transcription at 55°C for 20 min and denaturation at 95°C for 2 min. Amplification for 45 cycles: 95°C for 15 s, 55°C for 45 s, and 72°C for 15 s. Quality assurance was conducted at the National Institute of Virology, Pune. Samples that did not amplify were referred to the state-level Virus Research and Diagnostic Laboratory for further virological study.

### 2.8. Statistical Analysis

The statistical analysis of the data was conducted using SPSS Version 23. Continuous variables were evaluated by calculating means and standard deviations (SDs), while categorical variables were analyzed by determining frequencies and percentages. The chi-squared test was used to investigate associations between categorical variables, with significance set at a *p* value < 0.05.

## 3. Results

### 3.1. Diagnosis of Dengue Cases Using ELISA

A total of 2223 patients were enrolled, and blood samples were collected; among them, 1339 were from IPD and 884 from OPD. The data are presented by month, categorized by age groups ranging from 0 to > 61 years, with statistically significant differences identified at p<0.05 for each variable. 2041 patients (with less than 5 days of fever) were subjected to the NS1 test. Among them, 55 (2.7%) were NS1 positive patients, in which 33 were male (60%) and 22 were female (40%). However, we subjected the IgM test to samples from 182 patients who had fever for more than five days. Among them, 23 (12.63%) patients were IgM positive, in which 12 were male (52.17%) and 11 were female (47.8%). Overall, 78 (3.51%) patients were positive, including 45 males (57.69%) and 33 females (42.30%), as shown in Table 1. These positive cases were classified as warning signs with symptoms of abdominal pain, abdominal tenderness, continuous pain, persistent vomiting, restlessness, and nonwarning signs as per revised WHO guidelines. 51 NS1 and 23 IgM positive cases were recorded as without warning signs, while 4 NS1 and 2 IgM positive cases were recorded with warning signs as shown in Table 2. The age-wise analysis revealed that patients between 0–20 and 21–60 years age group were affected by dengue at the rate of ∼3.55 and ∼3.59%, while the patients above 60 years age group were affected by dengue at the lower rate of 2.38%. Data are tabulated in Table 3.

### 3.2. Season-Wise Positivity of Dengue Cases

**Figure 1:**
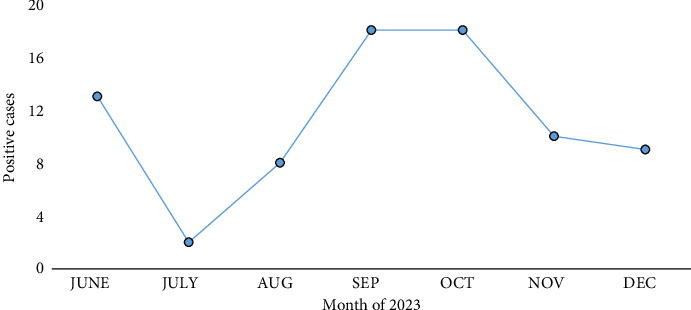
Month-wise distribution of dengue cases, reported between June and December 2023, and the peak was reported in September and October.

The seasonal analysis showed that the infection of dengue was highest in September and October months, where 36 (48.64%) cases were confirmed, followed by June at 13 (17.57%), November at 10 (13.51%), December at 9 (12.16%), August at 8 (10.81%), and July at 2 (2.7%).  Figure 1 displays the graphical view.

### 3.3. Serotyping of the NS1 Positive Cases at the Molecular Level

**Figure 2:**
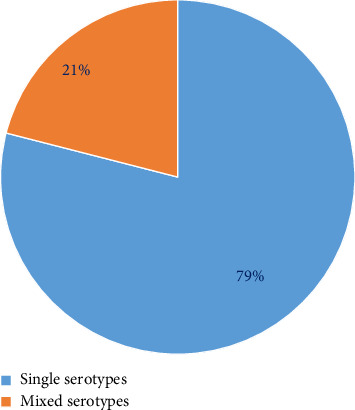
Prevalence of NS1 positive cases with single and mixed serotypes.

**Figure 3:**
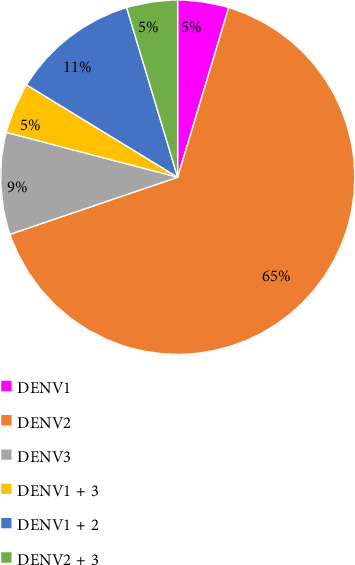
NS1 positive cases serotyped and detection of distribution of the specific serotypes singly and in combination.

Fifty-five NS1 positive cases were validated at the molecular level by RT-PCR and are tabulated in Table 4. Of those, 43 (78.18%) NS1 positive cases were successfully serotyped, which means that DENV RNA was detected. Twelve (21.82%) cases, on the other hand, are still untypable, which means that DENV RNA has not been detected. In-depth analysis revealed that 34/43 (79%) serotyped cases showed infection with a single serotype only; however, 9/43 (21%) serotyped cases showed infection with dual serotypes. Further analysis revealed that among the single serotype infections, DENV serotype 2 (65.11% of cases) was most prevalent, followed by DENV serotype 3 (9.30% of cases). However, in dual serotype infections, DENV serotypes 1 + 2 (11.62%) were most prevalent. Table 4 and Figures 2 and 3 present the data. Table 4 shows that the DENV-2 serotype (singly or in combination) is most frequent in 20–60-year-old patients. However, combinations of DENV mixed serotypes (1 + 2) have shown more frequency as compared to others.

## 4. Discussion

Dengue prevalence is continuously increasing worldwide over the past twenty years and posing a significant threat to public health. Dengue is also associated with hemorrhagic fever, with high morbidity and mortality rates, and presents with a spectrum of clinical signs ranging from mild to severe. As per WHO, due to the complex pathophysiological, ecological, and financial factors, the overall number of cases recorded worldwide increased 10-fold, from 500,000 to 5.2 million between 2000 and 2019 [15, 16]. An increase in dengue cases may be linked to rising urbanization and changes in vector behavior. Early diagnosis by detection of NS1Ag and IgM in the acute phase and the strengthening of vector control measures are most essential to prevent future dengue outbreaks. Due to the unavailability of a vaccine, vector control measures are the most effective way to reduce dengue prevalence and transmission. However, detection of serotypes is crucial to determine the virulence and severity of the disease mediated by the specific serotypes.

In the present study, data have shown that no severity of dengue cases (even in dengue cases with warning sign) or deaths were reported, which is likely due to the ongoing effective mosquito control program by the State Infectious Disease Control Program workers. In Bastar district, males were observed to be at higher risk of infection, possibly because it was noted that males, who typically work outdoors, often rest at home in minimal clothing, and probably expose themselves to be bitten by *Aedes* mosquitoes. Males tend to spend more time outdoors, especially during peak mosquito activity times (early morning and late night), and may differ in their use of protective measures such as mosquito nets, repellents, and long-sleeved clothing. However, males were potentially being less consistent in their use of these control measures. Earlier reports suggested that males are at a higher risk of dengue infection as per regional dengue investigations reports [17, 18]. A systematic review (based on 43 studies) reported that males are more prone to get dengue infections [19]. Male predominance of dengue infections was also reported in two investigations conducted in Uttarakhand and Uttar Pradesh, respectively [20, 21], which are similar to our report. Dengue infections were observed across different age groups, but no cases were found in children below 1 year of age in our study. Among the dengue cases, six exhibited dengue warning signs, while 72 cases were reported without any warning signs. Our findings highlight that the majority of dengue infections occurred between the months of July to September, due to the heavy rainfall in this area that created stagnant water bodies, which is the favorable environment for the vector and can lead to a rapid increase in populations.

RT-PCR-based molecular serotyping has shown that viral RNA was amplified in 43 samples (78.18%) among 55 dengue NS1-positive samples, while 12 samples (21.82%) remain untypable, which is possibly due to errors in recording the duration of fever, as virus detection usually does not occur in samples in which duration of fever exceeds five days. Among the 43 serotyped samples, 34 (79%) have shown the infection with a single serotype only, while 9 (21%) have serotyped with dual/mixed/coinfection. Among the mixed/coinfections, DENV1 + 2 serotypes were most prevalent and found in five samples, which is similar to previous published reports [22]. The presence of mixed/coinfections is concerning because it may lead to severe forms of dengue.

A report of the National Center for Vector Borne Diseases Control, India, since 2018 reveals the prevalence of dengue in different states. In respect of Chhattisgarh state, the maximum cases of dengue were seen in 2022 with 2679 cases and 10 deaths. State-wise observation has shown that the highest 67,271 cases were in West Bengal during 2022 and the highest 96 death cases were reported in Rajasthan during 2021 [23]. The dengue prevalence report of Haripur district, Khyber Pakhtunkhwa, Pakistan, led to 716 confirmed cases, where 421 male and 295 female cases were found between August and November, 2021. Age-wise analysis also revealed that individuals in the 16–30 age group were the most affected, which aligns with our findings reported in this study. The positive IgG was also reported highest in this age group with 244 confirmed antibodies [24].

Most dengue cases were reported among children aged 0 to 15, with a higher incidence in males than females, resulting in a male-to-female ratio of 1.8:1. The investigation revealed an increase in dengue cases starting in August 2014 and ending in July 2015, peaking in October before declining. This study also revealed that cases of dengue are more prevalent during the monsoon and post-monsoon seasons, as was similar to the previous report [25]. In the districts of Korea and Narsinghpur, 37.3% and 59% of cases were found to be positive for DF, respectively. RT-PCR analysis identified DENV serotype 1 genotype III as the major cause. This was the first recorded outbreak involving DENV serotype 1 in Central India [26]. In 2023, Dhaka accounted for approximately 48% of recorded dengue cases and 74% of dengue-related deaths, leading to a higher case fatality rate of 0.74%, compared to 0.24% in the rest of the country. However, the distribution of cases has changed over time. Comprehensive, countrywide geographic surveillance, coupled with the collection of clinical, demographic, and socioeconomic data, is crucial to better understand age and gender-related variations in morbidity and mortality, as well as the characteristics of DENV transmission hotspots [27].

The DENV typically leads to fever outbreaks, commonly observed in impoverished urban regions. However, its prevalence is not limited to these areas; it also affects affluent tropical and subtropical regions, suburban zones, and rural settings. Various factors, including the degree of urbanization, temperature fluctuations, humidity levels, rainfall intensity, and the effectiveness of vector control measures, contribute to the vulnerability of an area that may be prone to outbreak. As part of addressing this issue, it is crucial to raise awareness among local communities regarding the risk factors associated with DENV transmission and the potentially severe consequences of dengue infection. Organizing awareness seminars and workshops plays a vital role in disseminating this information widely. To launch an effective campaign aimed at educating the public on preventive measures against DF, the involvement of healthcare professionals is indispensable. Their specialized knowledge and skills equip them to lead such initiatives effectively.

The present study has several limitations: (i) it specifically focuses on the endemic district of Bastar in Chhattisgarh, (ii) the sample size is small, covering only a six-month period (June 2023–December 2023), (iii) there may be selection bias, (iv) asymptomatic or mild cases that did not seek healthcare could have been missed, (v) although the study is hospital-based, we cannot be certain that the serotype distribution in the community fully reflects our findings, (vi) since we did not use IgG antibodies, we could not differentiate between primary and secondary dengue cases, and (vii) diagnostic limitations arose as serotype distribution was determined by RT-PCR rather than the PRNT assay recommended by the WHO, which was not available in the laboratory. Consequently, generalizing the findings to the entire state or nation is not feasible. Differences in patient demographics, seasonal variations, or diagnostic sensitivities are the key potential sources of variability for dengue prevalence and circulating serotypes of dengue. A long-term study is warranted, to comprehensively assess seasonal variations and other risk factors influencing DENV prevalence in Bastar district.

## 5. Conclusion

DF is a mosquito-borne viral infection that presents a significant global health threat. Its prevalence has been raised in numerous regions worldwide, attributed to factors like urbanization, climate change, and inadequate mosquito control measures. It is advisable to refer to the latest updates from health organizations such as the National Vector Borne Disease Control Program or relevant public health authorities for the most current and region-specific data on DENV prevalence. This study revealed that DENV2 is most prevalent in Bastar district, with a higher incidence observed in adult (20–60 years age group) individuals. Dengue severity cases may arise from mixed infections, where patients are infected with two or more different serotypes of the DENV. The study's serotyping data might signify the early detection and identification of circulating serotypes, which provides valuable insights to clinicians for the management of dengue infections. DENV mixed serotypes, specifically DENV1 + 2, have shown most frequency compared to others, reflecting the severe dengue outbreak in the endemic region of Bastar district, Chhattisgarh. Hence, continuous epidemiological surveillance of DENV in the regional area is essential to anticipate future heterologous infections and their impact on healthcare and to validate the serotype implications for dengue outbreaks. Early detection and vigilant monitoring of patients are crucial for identifying the circulating serotypes of DENV, facilitating subsequent epidemiological studies and disease control strategies.

## Figures and Tables

**Table 1:** Diagnosis of dengue using fever as a major parameter for detection of NS1 antigen and IgM antibody.

Major parameter (fever)	Test used	Sample tested *n* (%)	Negative cases *n* (%)	Positive cases *n* (% of positivity)	Positive Male, *n* (%)	Positive Female, *n* (%)
< 5 days	NS1 Ag	2041/2223 (91.81)	1986/2041 (97.30)	55/2041 (2.70)	33/55 (60)	22/55 (40)
> 5 days	IgM Ab	182 (8.19)	159/182 (87.36)	23/182 (12.63)	12/23 (52.17)	11/23 (47.82)
Grand total		2223	2145/2223 (96.49)	78/2223 (3.51)	45/78 (57.70)	33/78 (42.30)

**Table 2:** Distribution of dengue severity in NS1 and IgM positive patients (based on symptoms cited in revised WHO classification system).

Testing parameters	Total number of dengue positive cases, *n* (%)	Dengue without warning sign, *n* (%)	Dengue with warning sign, *n* (%)
Dengue NS1Ag	55/78 (70.51)	51/55 (92.72)	4/55 (7.27)
Dengue IgM Ab	23/78 (29.49)	21/23 (91.30)	2/23 (8.70)
Grand total	78	72/78 (92.30)	6/78 (7.70)

**Table 3:** Age-wise distribution of confirmed dengue cases in patients between 0 and above 61 years of age.

Age group	Enrolled samples *n*	Negative *n* (%)	Positivity *n* (%)
0–20	479	462/479 (96.45)	17/479 (3.55)
21–60	1618	1560/1618 (96.41)	58/1618 (3.59)
Above 61	126	123/126 (97.62)	3/126 (2.38)
Total	2223	2145/2223 (96.49)	78/2223 (3.51)

**Table 4:** Frequency and distribution of DENV serotypes in patients of different age groups.

Age group (years)	NS1 positive samples (*n*)	Samples remain untypable	Samples serotyped	Single serotypes detected			Coinfections or mixed serotypes detected			Total serotypes/total sample
				DENV 1	DENV 2	DENV 3	DENV 1 + 3	DENV 1 + 2	DENV 2 + 3	
0–20	9	5	4	1	1	0	1 (1 + 3)	1 (1 + 2)	0	6/4
21–60	45	7	38	1	26	4	1 (1 + 3)	4 (1 + 2)	2 (2 + 3)	45/38
> 60	1	0	1	0	1	0	0	0	0	1/1
Total	55	12/55 (21.82)	43/55 (78.18)	2/43 (4.65%)	28/43 (65.11%)	4/43 (9.30%)	2/43 (4.65%)	5/43 (11.62%)	2/43 (4.65%)	52/43
Grand total	55	12	43	34/43 (79%)			9/43 (21%)			

## Data Availability

The data used to support the findings of this study are included within the article.
